# Data on left ventricular expression of STAT3 and AKT in transgenic mouse models with B16F10 melanoma

**DOI:** 10.1016/j.dib.2019.104508

**Published:** 2019-09-18

**Authors:** Stefan Pietzsch, Melanie Ricke-Hoch, Britta Stapel, Denise Hilfiker-Kleiner

**Affiliations:** a Department of Cardiology and Angiology, Hannover Medical School, Hannover, Germany; b Department of Psychiatry, Social Psychiatry and Psychotherapy, Hannover Medical School, Hannover, Germany

**Keywords:** Heart failure, Cancer, AKT, STAT3

## Abstract

The dataset describes protein expression of phosphorylated and total signal transducer and activator of transcription 3 (STAT3), protein kinase B (AKT) and suppressor of cytokine signalling 3 (SOCS3) in left ventricular tissue (LV) from healthy and B16F10 melanoma tumour-bearing (B16F10-TM) wildtype (WT) mice, mice with cardiomyocyte-specific constitutively active AKT transgene (AKTtg) and mice with cardiomyocyte-restricted deletion of STAT3 (CKO) analysed in Western blot and/or fluorescence microscopy experiments. The data presented in this article are related to the research paper entitled “Modulation of cardiac AKT and STAT3 signalling in preclinical cancer models and their impact on the heart”, available in Biochim. Biophys. Acta Mol. Cell Res. (1).

**Table:** Specifications Table

Subject area	*Biology*
More specific subject area	*Molecular biology*
Type of data	*Images and densitometric quantification, figures*
How data was acquired	*Immunofluorescence microscopy, Western blot analyses, RT-PCR*
Data format	*analysed*
Experimental factors	*Samples from left ventricular tissue of mouse hearts from healthy or melanoma tumour-bearing wildtype C5*7BL*/6N mice, mice with cardiomyocyte-specific constitutively active AKT transgene and mice with cardiomyocyte-restricted deletion of STAT3*
Experimental features	*Male mice of genotypes described above were injected with B16F10 melanoma cells or PBS as a vehicle. Control and B16F1*0-TM *hearts were harvested when mice presented with advanced cancer.*
Data source location	*Experiments were conducted at MHH research facility in accordance with the German animal protection law and with European Communities Council Directive 86/*609/EEC *and 2010/63/EU for the protection of animals used for experimental purposes. All experiments were approved by the local institutional animal care and research advisory committee and permitted by LAVES (Niedersächsisches Landesamt für Verbraucherschutz und Lebensmittelsicherheit; Oldenburg, Lower Saxony, Germany)*
Data accessibility	*data is included in this article; raw data is included in supplementary file*
Related research article	*Data in this article are related to the research paper:* Pietzsch S, Ricke-Hoch M, Stapel B, Hilfiker-Kleiner D. Modulation of cardiac AKT and STAT3 signalling in preclinical cancer models and their impact on the heart. Biochim Biophys Acta Mol Cell Res. 2019. https://doi.org/10.1016/j.bbamcr.2019.07.014. [Epub ahead of print]

**Table:** 

**Value of the data** • The data show B16F10 melanoma cancer-induced changes in left ventricular tissue protein expression of key cardiac signalling molecules STAT3 and AKT in WT mice and demonstrate which of these changes are persistent in genetically modified mice • The data could be useful to further understand and explore the role of cardiac AKT activation during cancer-induced cardiac atrophy • Data could be useful to further explore the role of cancer-induced cardiac STAT3 activation associated with cardiac atrophy and to elucidate in which cardiac cell type the STAT3 activation is more relevant with regards to development of cardiac atrophy in this context

## 1 Data

Mice bearing severe B16F10 melanoma tumours (B16F10-TM) develop cardiac atrophy at an advanced tumour disease stage when cancer-induced cachexia indicated by body weight loss of 10–15% compared to healthy tumour-free control mice is present [1], [2]. This is associated with loss of cardiac function and substantial cardiac molecular and metabolic alterations and high mortality [1], [2]. Among the molecular alterations reported to date are reduced phosphorylation of protein kinase B (AKT) and upregulated ubiquitin proteasomal system (UPS), and autophagy [2]. In addition, further key cardiac signalling pathways were affected by B16F10 tumour burden including constitutive high activation of signal transducer and activator of transcription 3 (STAT3), and reduction of mitogen-activated protein kinase p38 (p38) and mitogen-activated protein kinase p44/42 [1]. Impaired systemic insulin signalling by the growing tumour accounted for part of these impairments, i.e. reduced left ventricular (LV) function, reduced phosphorylation of AKT, enhanced UPS and autophagy, as well as reduced cardiac glucose uptake [2]. To further evaluate the role of tumour-induced alterations in cardiac signalling, B16F10 melanoma tumours were also induced in mice with either a cardiomyocyte-specific constitutive activation of AKT (AKTtg) or in mice with a cardiomyocyte-specific deletion of STAT3 (CKO).

**Fig. 1:**
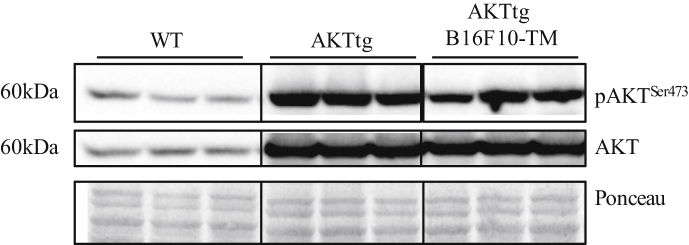
**A)** Representative Western blots depicting protein levels in left ventricular (LV) tissue from hearts of healthy wildtype (WT) mice, mice with cardiomyocyte-specific constitutively active AKT transgene (AKTtg) and tumour-bearing (B16F10-TM) AKTtg (n = 5 each) of phosphorylated and total protein kinase B (AKT) and Ponceau S stain as loading control; Frames indicate cropping of Western blot images for presentation.

We observed that overexpression of constitutively activated AKT attenuated tumour-induced cardiac dysfunction and cardiac atrophy [1]. In addition, we showed that AKTtg was able to correct the expression of markers for impaired UPS and autophagy [1]. Here we show levels of phosphorylated AKT (Ser473) and total AKT protein in left ventricular tissue of tumour-free wildtype (WT) control mice, tumour-free AKTtg and AKTtg B16F10-TM which reveal that tumour disease did not reduce total and phosphorylated AKT (Fig. 1A).

**Fig. 2:**
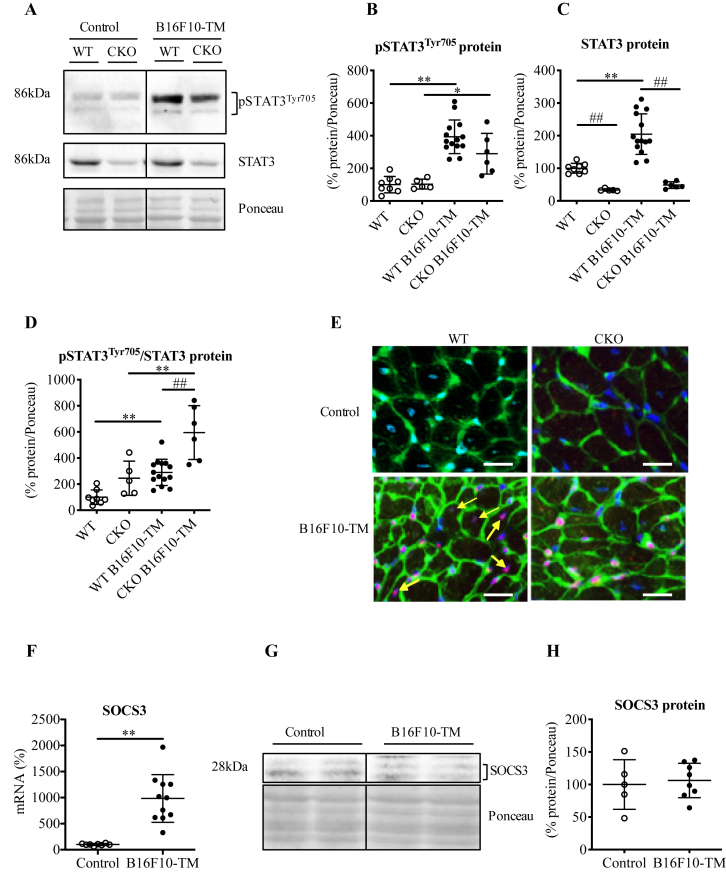
**A)** Representative Western blots depicting protein levels in LV tissue from hearts of healthy and B16F10-TM WT mice (n = 8–14) and mice with cardiomyocyte-restricted deletion of signal transducer and activator of transcription 3 (STAT3) (CKO, n = 5–6) of phosphorylated and total STAT3 and Ponceau S stain as loading control; **B-D)** Quantification of pSTAT3, STAT3 and pSTAT3/STAT3 ratio based on Western blots presented in (A) normalized on Ponceau S staining; **E)** Representative images of immunofluorescence staining of LV tissue from hearts of healthy and B16F10-TM WT and CKO (n = 5 each) showing phosphorylated STAT3 (red) wheat-germ-agglutinin (WGA, green) and 4′,6-diamidino-2-phenylindole (DAPI, blue), scale bars indicate 20 μm; arrows indicate pSTAT3 positive nuclei in cardiomyocytes of WT B16F10-TM which is not present in cardiomyocytes of CKO B16F10-TM **F)** Quantitative mRNA levels (qRT-PCR) normalized on 18S expression of suppressor of cytokine signalling 3 (SOCS3) in healthy WT (control, n = 8) and B16F10-TM (n = 11); **G)** Representative Western blots depicting protein levels in LV tissue from hearts of healthy WT (n = 5) and B16F10-TM (n = 8) of SOCS3 and Ponceau S stain as loading control; **H)** Quantification of (G) normalized on Ponceau S staining. Data are depicted as mean ± SD with WT mean (B–D) respectively control group (F, H) mean defined as 100%; *p < 0.05, **p < 0.01 vs. respective tumour-free control and ##p < 0.01 vs. respective WT, using either 2-way ANOVA with Bonferroni posttest (B–D) or 2-tailed Student's unpaired t tests (F, H) with or without Welch's correction as required. Frames indicate cropping of Western blot images for presentation.

In contrast, the cardiomyocyte-restricted deletion of STAT3 did not prevent tumour-induced cardiac dysfunction or the cardiac atrophy phenotype [1]. Interestingly, it did also not lead to a worsening of the tumour-induced cardiac dysfunction and atrophy, a finding that was unexpected since STAT3 is an important factor for cardioprotection [3], [4], [5], [6]. Here, we present levels of phosphorylated STAT3 (Tyr705) and total STAT3 protein in LV tissue of tumour-free (control) and B16F10 bearing WT and CKO mice (Fig. 2A–D). While tumour disease did alter total STAT3 protein levels in WT mice, the ratio of phosphorylated STAT3 to total STAT3 was higher in tumour diseased CKO mice, suggesting that non-cardiomyocytes display a higher STAT3 activation state compared with WT B16F10-TM (Fig. 2B–E). In order to analyse in which cardiac cells tumour disease induces STAT3 activation, we performed immunofluorescence analyses of STAT3 (Tyr705) phosphorylation combined with wheat-germ agglutinin (WGA) in WT and CKO mice with and without B16F10 tumours. This co-staining showed localization of low phosphorylated STAT3 in LVs of healthy control mice from both genotypes and enhanced staining for phosphorylated STAT3 in cardiomyocytes and non-myocytes (distinguished by cell size and morphology) in WT mice with B16F10-TM, while in tumour diseased CKO mice only non-myocytes stained positive for phosphorylated STAT3 (Fig. 2E).

Suppressor of cytokine signalling 3 (SOCS3) is a transcriptional target of STAT3 completing a negative feedback loop after activation of the glycoprotein 130 (gp130) receptor by interleukin (IL)-6 family cytokines [7]. In fact, SOCS3 binds to the SHP domain of the gp130 receptor and terminates activation of gp130/JAK2/STAT3 signalling [7]. As expected, we observed upregulated SOCS3 mRNA levels in WT B16F10-TM as the result of activated STAT3, while SOCS3 protein levels were not upregulated (Fig. 2F–H). While this feature explains the constitutive activation of STAT3, we do not know so far why SOCS3 mRNA is not translated or alternatively why SOCS3 protein is rapidly degraded in the heart of B16F10-TM.

Raw data for all figures is included in supplementary file.

## 2 Experimental design, materials and methods

Data were obtained from left ventricular tissue (LV) of hearts from male mice (12 ± 2 weeks of age). All mice were of C57BL/6N background. The B16F10 tumour cells were from mice of the same background. Characterization of transgenic mice with a cardiomyocyte-specific knockout of STAT3 (αMHC-Cre^tg/-^; STAT3^fl/fl^; CKO) [3], [7] or constitutively active AKT transgene (αMHC-Akt^tg/-^: CAkt^tg^; AKTtg) and AKTtg [8], [9] has been described previously.

Male mice were injected with B16F10 melanoma cells (1 × 10^6^) or PBS. After cell injection, tumour-bearing mice and tumour-free controls received Novalgin (Zentiva), 1000 mg/kg/day in drinking water. Mice were housed in groups of five and maintained on a 14 h/10 h light/dark cycle with standard laboratory chow and water freely available. Control and B16F10-TM hearts were harvested, cut in half, and snap-frozen in liquid nitrogen or embedded in OCT and stored at −80 °C when mice with advanced cancer presented with body weight reduction of 10–15% compared with age-matched tumour-free controls. Animal health condition was assessed based on the guidelines of recognition of distress in experimental animals as proposed by Morton and Griffith [10]. All animal studies were in accordance with the German animal protection law and with European Communities Council Directive 86/609/EEC and 2010/63/EU for the protection of animals used for experimental purposes. All experiments were approved by the local institutional animal care and research advisory committee and permitted by LAVES (Niedersächsisches Landesamt für Verbraucherschutz und Lebensmittelsicherheit; Oldenburg, Lower Saxony, Germany).

Immunofluorescence staining was carried out in LV cryosections. For pSTAT3 (T705) staining pSTAT3 (Y705) (D3A7) XP from Cell Signaling Technologies (CST, #9145) was used. Cryosections were permeabilized with methanol, blocked with donkey serum and incubated with primary antibody overnight. After washing with PBS, secondary Cy3-conjugated anti-rabbit-antibody from Jackson (#711-165-152) was applied at room temperature. For counterstaining WGA (Vector) was used. After washing UltraCruz mounting medium containing DAPI from Santa Cruz (sc-24941) was applied.

Total RNA from adult murine hearts was isolated with Trizol (Invitrogen) and cDNA synthesis was performed as described previously [5], [11]. Real-time PCR with SYBR green dye method (Brilliant SYBR Green Mastermix-Kit, Thermo Fisher) was performed with the AriaMx Real-Time PCR System (Agilent Technologies) as described previously [2]. Semi-quantitative analyses were based on normalization to 18S expression with ΔΔCt-method. Mean of control group was defined as 100%. The following primers were used:

**Table:** 

Target	Sense primers (5′ to 3′)	Antisense primers (5′ to 3′)
*18S rRNA*	GTAACCCGTTGAACCCCATT	CCATCCAATCGGTAGTAGCG
*SOCS3 mRNA*	ATGGTCACCCACAGCAAGTTT	TCCAGTAGAATCCGCTCTCCT

Protein expression levels were determined by Western blotting, using SDS PAGE as previously described [2]. The following antibodies were used: pSTAT3 (T705) Cell Signaling Technologies (CST, #9145); STAT3 (CST, #9139); pAKT (S473) (CST, #4060); AKT (CST, #9272); SOCS3 antibody (M − 20, Santa Cruz, sc-7009).

## Funding

This study was supported by the DFG/KFO311; DFG-Grants: HI-842/9-1; RI-2531/1-1 and Stiftung Gerdes. Funding sources did not have any involvement in the study design, collection, analysis and interpretation of data, the writing of the report or the decision to submit the article for publication.
