# Supplementary material for: Data on left ventricular expression of STAT3 and AKT in transgenic mouse models with B16F10 melanoma
Source: Data Brief. 2019 Sep 18;26:104508. doi: 10.1016/j.dib.2019.104508 (PMC6811954; doi:10.1016/j.dib.2019.104508)
Supplement: Multimedia component 1 [file mmc1.docx]

Supplementary file: Raw/primary data

Raw data for Figure 1: raw Western blot images that were cropped for presentation in Figure 1

Raw data for Figure 2: raw Western blot images that were cropped for presentation in Figure 2A

Raw data Figure 2B:

Raw densitometric single value per sample normalized to Ponceau in % with WT mean as 100 %

| **WT** | **CKO** | **WT B16F10-TM** | **CKO B16F10-TM** |
| --- | --- | --- | --- |
| 29,56 | 87,43 | 350,78 | 374,81 |
| 77,45 | 84,48 | 609,64 | 485,23 |
| 192,99 | 74,58 | 365,63 | 236,15 |
| 134,96 | 146,37 | 467,61 | 154,11 |
| 60,40 | 123,36 | 255,36 | 184,61 |
| 75,67 |  | 337,14 | 302,17 |
| 118,31 |  | 574,26 |  |
| 110,66 |  | 259,57 |  |
|  |  | 330,58 |  |
|  |  | 374,43 |  |
|  |  | 337,77 |  |
|  |  | 448,00 |  |
|  |  | 393,17 |  |
|  |  | 404,80 |  |

Raw data Figure 2C:

Raw densitometric single value per sample normalized to Ponceau in % with WT mean as 100 %

| **WT** | **CKO** | **WT B16F10-TM** | **CKO B16F10-TM** |
| --- | --- | --- | --- |
| 82,40 | 32,11 | 232,78 | 47,74 |
| 127,15 | 32,00 | 183,82 | 57,97 |
| 90,46 | 39,79 | 196,43 | 62,51 |
| 105,75 | 30,45 | 166,07 | 35,24 |
| 96,87 | 32,78 | 281,43 | 42,67 |
| 82,54 |  | 122,03 | 43,93 |
| 111,07 |  | 180,86 |  |
| 103,77 |  | 288,19 |  |
|  |  | 267,84 |  |
|  |  | 311,70 |  |
|  |  | 183,24 |  |
|  |  | 149,85 |  |
|  |  | 117,58 |  |
|  |  | 183,49 |  |

Raw data Figure 2D:

Raw densitometric single value per sample normalized to Ponceau in % with WT mean as 100 %

| **WT** | **CKO** | **WT B16F10-TM** | **CKO B16F10-TM** |
| --- | --- | --- | --- |
| 35,00 | 132,00 | 151,00 | 788,86 |
| 59,00 | 127,00 | 333,00 | 841,08 |
| 206,00 | 225,00 | 161,00 | 379,57 |
| 77,00 | 442,00 | 180,00 | 349,31 |
| 87,00 | 300,00 | 237,05 | 544,69 |
| 158,00 |  | 237,88 | 665,39 |
| 81,00 |  | 252,50 |  |
| 97,00 |  | 290,29 |  |
|  |  | 348,00 |  |
|  |  | 358,00 |  |
|  |  | 522,01 |  |
|  |  | 359,94 |  |
|  |  | 368,01 |  |
|  |  | 257,74 |  |

Raw data Figure 2E:

Raw data Figure 2F:

Raw 18S normalized single value per sample in % with Control mean as 100 %

| Control | B16F10-TM |
| --- | --- |
| 97,86 | 329,16 |
| 102 | 609,99 |
| 87,59 | 997,83 |
| 114,8 | 1254,28 |
| 64,56 | 672,15 |
| 107,1 | 1968,18 |
| 95,18 | 1254,28 |
| 130,9 | 618,51 |
|  | 1335,02 |
|  | 1018,79 |
|  | 761,47 |

Raw Western blot images that were cropped for presentation in Figure 2G

Raw data Figure 2H:

Raw densitometric single value per sample normalized to Ponceau in % with Control mean as 100 %

| Control | B16F10-TM |
| --- | --- |
| 151,25 | 135,12 |
| 99,96 | 98,65 |
| 84,81 | 126,04 |
| 48,24 | 115,05 |
| 115,74 | 137,41 |
|  | 83,58 |
|  | 89,8 |
|  | 64,18 |
